# Supplementary material for: Lineage-specific protection and immune imprinting shape the age distributions of influenza B cases
Source: Nat Commun. 2021 Jul 14;12:4313. doi: 10.1038/s41467-021-24566-y (PMC8280188; doi:10.1038/s41467-021-24566-y)
Supplement: Supplementary file 1 — Supplementary information [file 41467_2021_24566_MOESM1_ESM.pdf]

Lineage-specific protection and immune imprinting shape the age distributions of influenza B cases

Marcos C. Vieira, Celeste M. Donato, Philip Arevalo, Guus F. Rimmelzwaan, Timothy Wood, Liza Lopez, Q. Sue Huang, Vijaykrishna Dhanasekaran, Katia Koelle, Sarah Cobey

Supplementary information

Supplementary Table 1. Parameter estimates from the New Zealand data compared to estimates based on additional data sets.

Parameter	New Zealand	EU [1]	EU [2]	Australia	China	Definition
β_1	0.09 (0.07-0.10)	0.11 (0.10-0.13)	0.07 (0.06-0.09)	0.13 (0.12-0.15)	0.10 (0.09-0.11)	Baseline annual infection probability for preschoolers (0-5 years old)
β_2	0.16 (0.13-0.20)	0.19 (0.17-0.23)	0.12 (0.10-0.14)	0.21 (0.18-0.25)	0.29 (0.26-0.33)	Baseline annual infection probability for people 6-17 years old
β_3	0.12 (0.09-0.15)	0.12 (0.10-0.16)	0.07 (0.06-0.10)	0.14 (0.11-0.18)	0.38 (0.35-0.49)	Baseline annual infection probability for people 18+ years old
χ_{VV}	0.89 (0.85-0.96)	1.00 (0.94-1.00)	0.96 (0.93-1.00)	0.95 (0.94-0.97)	1.00 (0.99-1.00)	Protection against B/Vic from any prior B/Vic infection
χ_{YY}	0.28 (0.1-0.43)	0.54 (0.40-0.68)	0.75 (0.62-0.82)	0.84 (0.79-0.88)	1.00 (0.98-1.00)	Protection against B/Yam from any prior B/Yam infection
γ_{YV}	0.00 (0.00-1.00)	0.97 (0.41-1.00)	1.00 (0.89-1.00)	1.00 (0.81-1.00)	0.94 (0.91-0.99)	Protection from B/Vic infection against B/Yam (as a fraction of χ_{YY})†
γ_{VY}	0.00 (0.00-0.07)	0.00 (0.00-0.23)	0.57 (0.00-0.77)	0.84 (0.79-0.88)	0.95 (0.93-0.96)	Protection from B/Yam infection against B/Vic (as a fraction of χ_{VV})†
R_V	0.85 (0.00-1.00)	(0.00-1.00)‡	0.00 (0.00-1.00)	0.00 (0.00-0.52)	(0.00-1.00)‡	Additional B/Vic protection if 1st infection was with B/Vic
R_Y	0.96 (0.87-1.00)	0.86 (0.76-0.93)	1.00 (0.89-1.00)	0.94 (0.61-1.00)	(0.00-1.00)‡	Additional B/Yam protection if 1st infection was with B/Yam
γ_{VA}	1.00 (0.93-1.00)	0.95 (0.94-1.00)	1.00 (0.95-1.00)	1.00 (0.98-1.00)	0.00 (0.00-0.31)	Protection against B/Vic from pre-1988 infections (as a fraction of χ_{VV})†
γ_{YA}	1.00 (0.00-1.00)	1.00 (0.00-1.00)	0.00 (0.00-0.40)	1.00 (0.79-1.00)	0.98 (0.97-1.00)	Protection against B/Yam from pre-1988 infections (as a fraction of χ_{YY})†
ρ	1.97 (1.73-2.26)	0.58 (0.5-0.68)	0.60 (0.52-0.73)	1.36 (1.20-1.54)	0.4 (0.33-0.5)	Reporting factor for children under 2 years old.

[1]Fit to European Union data assuming B/Yamagata was the only lineage circulating between 1988-2000. [2]Fit to European Union data assuming B/Victoria and B/Yamagata were the only lineages circulating in 1988-1993 and 1993-2000, respectively. †Cross-lineage protection does not apply when within-lineage protection is present. ‡ Flat likelihood surface.

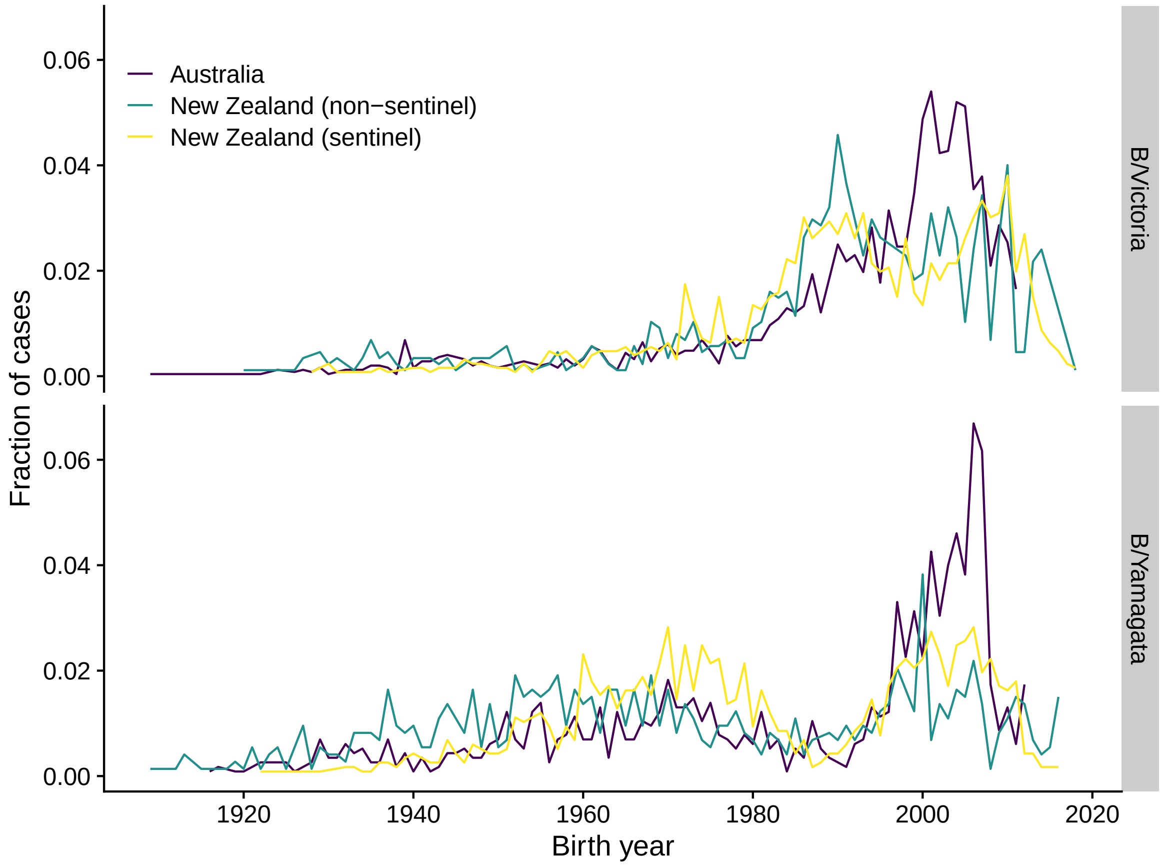

Supplementary Fig. 1. Distribution of medically attended influenza B cases in New Zealand (2001-2019) and Australia (2002-2013) by birth year of the patient. The fraction of cases in each birth year was calculated relative to all cases observed for each lineage (separately by type of surveillance in New Zealand).

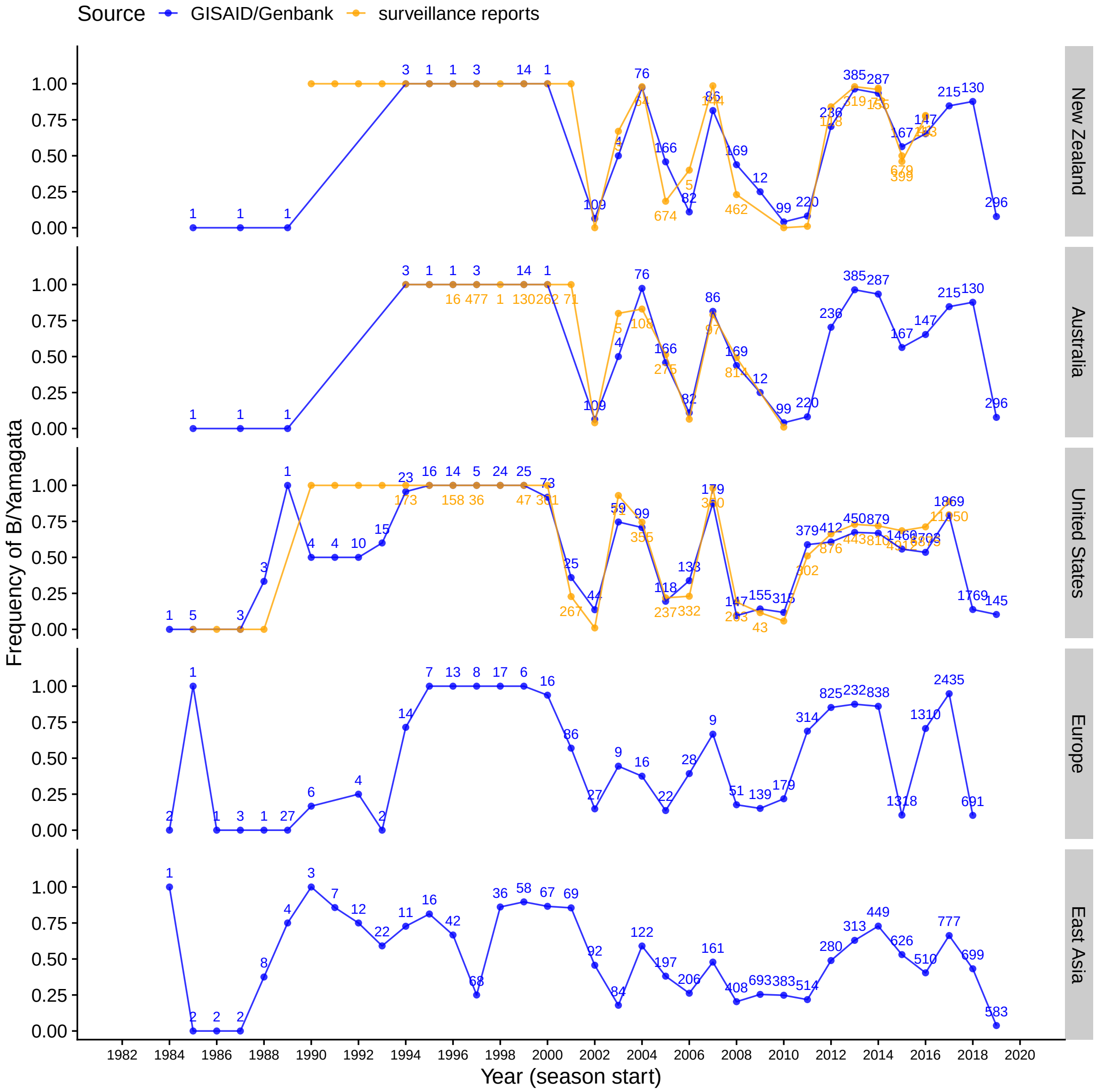

Supplementary Fig. 2. Comparison of lineage frequency estimates based on sequence data and surveillance reports. Frequencies are shown by annual influenza seasons, which span multiple calendar years in the Northern hemisphere but are contained in a single calendar year in the Southern Hemisphere (e.g., 2006 refers to the 2006/2007 influenza season in the United States, Europe and East Asia and to the 2006 season in Australia and New Zealand). Numbers indicate the number of isolates collected in that season and deposited on GISAID or the NCBI Influenza Virus Database (blue) or tested by surveillance (orange; the total number of isolates tested was not reported in some of the surveillance reports). Sequence database isolates from Australia and New Zealand were grouped together to estimate lineage frequencies.

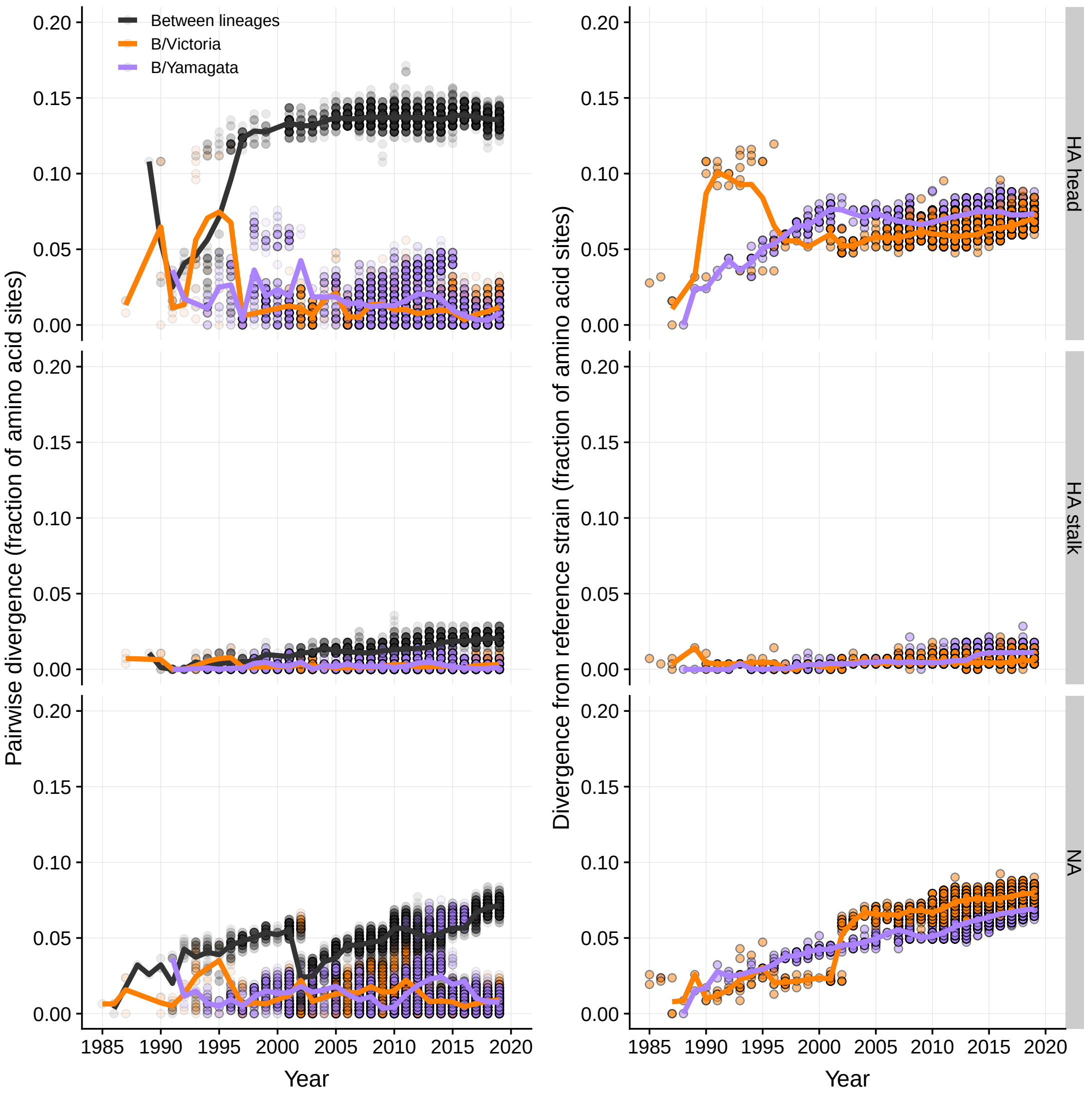

Supplementary Fig. 3. Amino acid divergence in the hemagglutinin (HA) and neuraminidase (NA) proteins within and between influenza B lineages. The left panel shows the fraction of amino acid sites that differ between pairs of sequences in each season (each point is a pair). Up to 500 randomly chosen pairs are shown, while the solid lines represent the annual average calculated from all pairs given two samples of up to 100 sequences from each lineage (i.e, up to 4,950 pairs). The right panel shows divergence of strains circulating at different times from reference strains B/Victoria/2/87 and B/Yamagata/16/88.

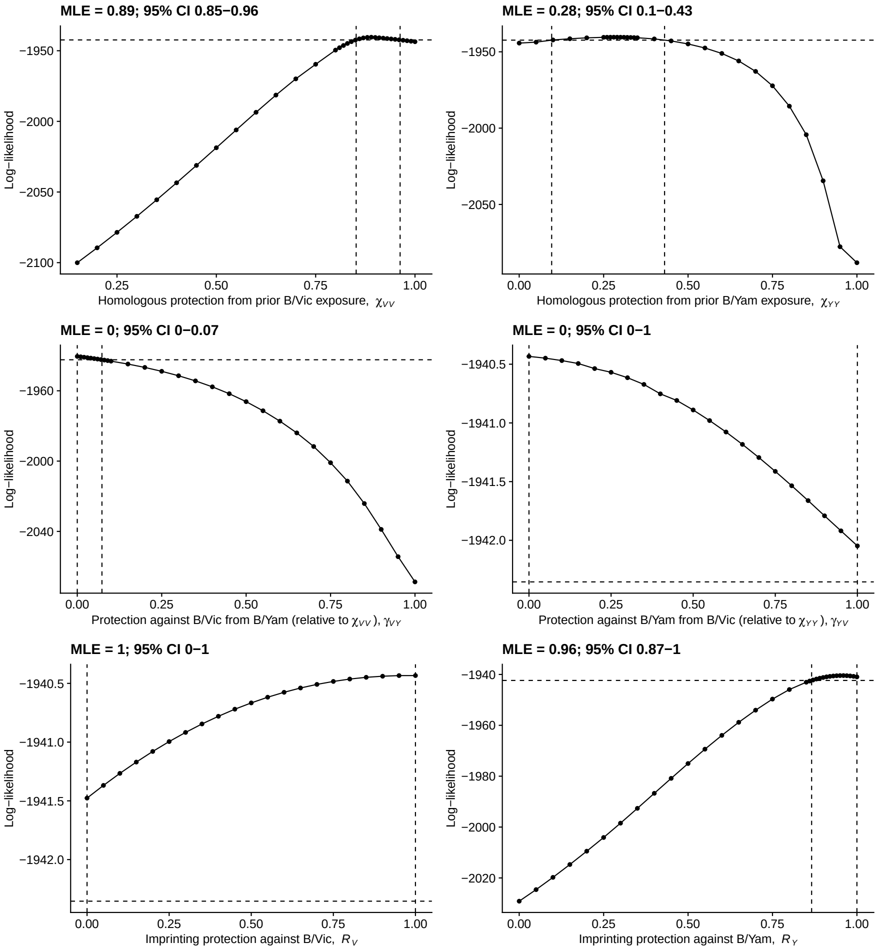

Supplementary Fig. 4. Likelihood profiles for protection parameters estimated from the complete New Zealand data. The 95% confidence interval based on a likelihood ratio test with one degree of freedom is indicated by the vertical dashed lines.

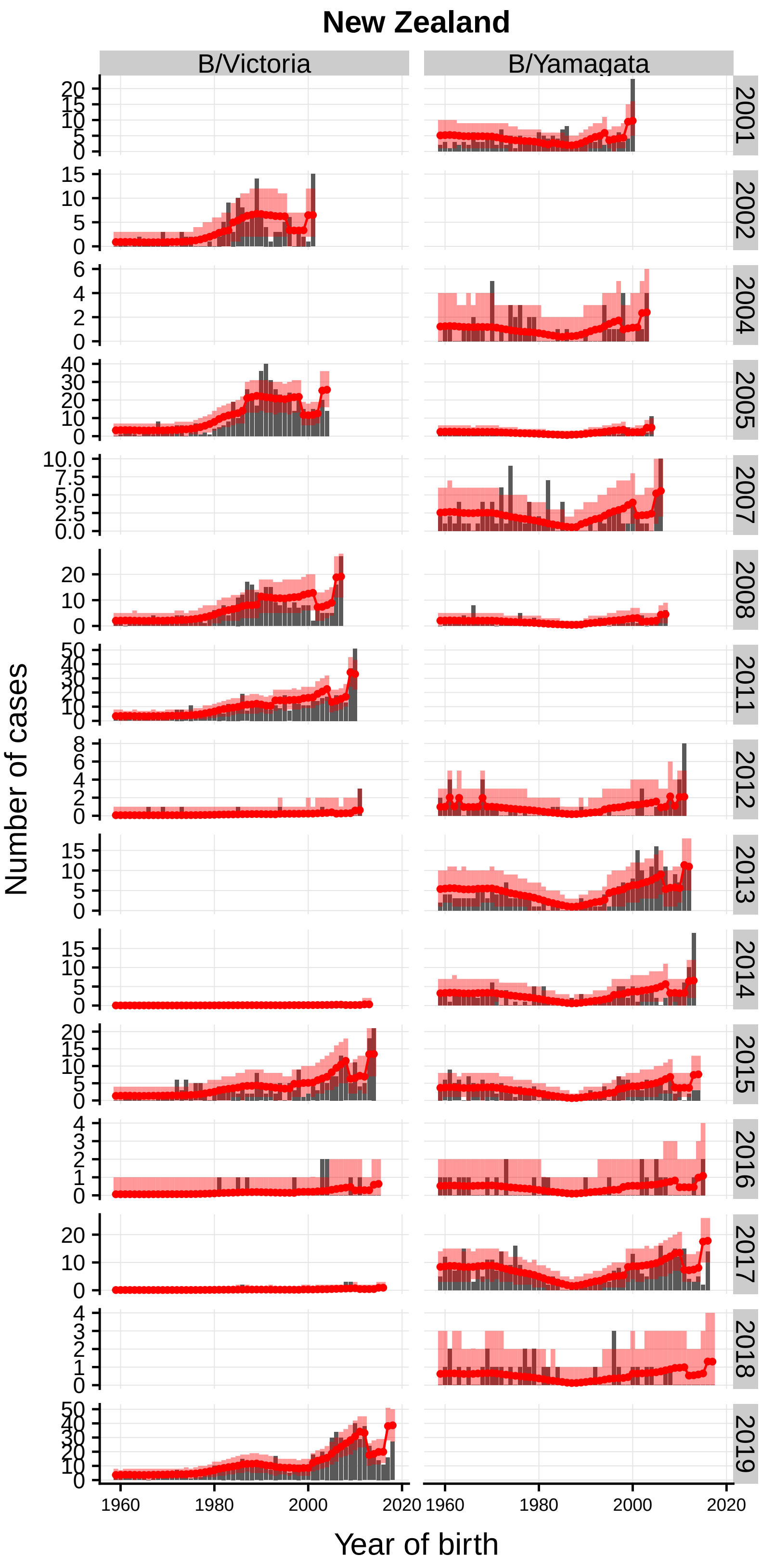

Supplementary Fig. 5. Observed and predicted distributions of influenza B cases in New Zealand by observation year. Red lines and dots show the fraction of cases in each birth cohort as predicted by the model. Vertical bars are 95% bootstrap confidence intervals based on $n = 1000$ multinomial draws from the predicted distributions indicated by the dots. Season/lineage combinations with fewer than 5 cases were omitted to improve visualization.

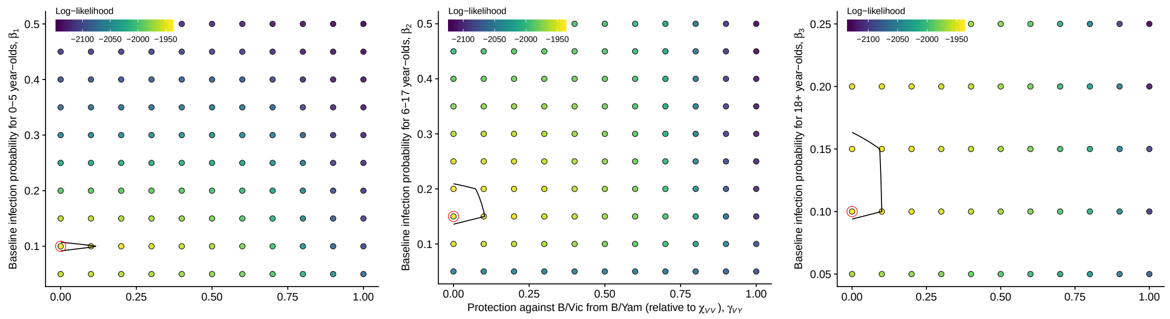

Supplementary Fig. 6. Bivariate profiles of age-specific baseline probabilities of infection and protection from B/Yamagata against B/Victoria. The red circle indicates the maximum likelihood value in the profile. The contour indicates the 95% confidence interval based on a bilinear interpolation of the profiled points.

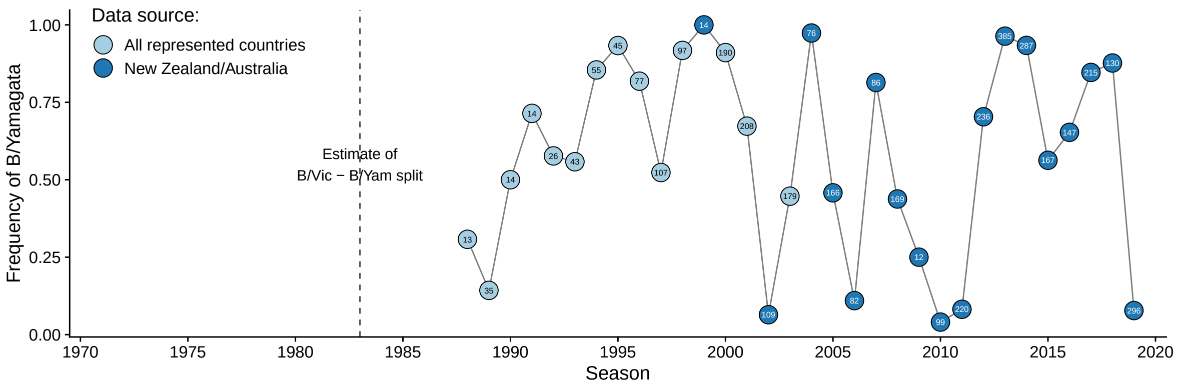

Supplementary Fig. 7. Lineage frequencies used in sensitivity analysis to test the effect of having only B/Yamagata in New Zealand in the 1990s. If fewer than 10 isolates were reported in New Zealand and Australia combined in a season, we estimated frequencies using isolates from all countries represented in the databases. The number inside each circle indicates the number of isolates used to estimate lineage frequencies in each season.

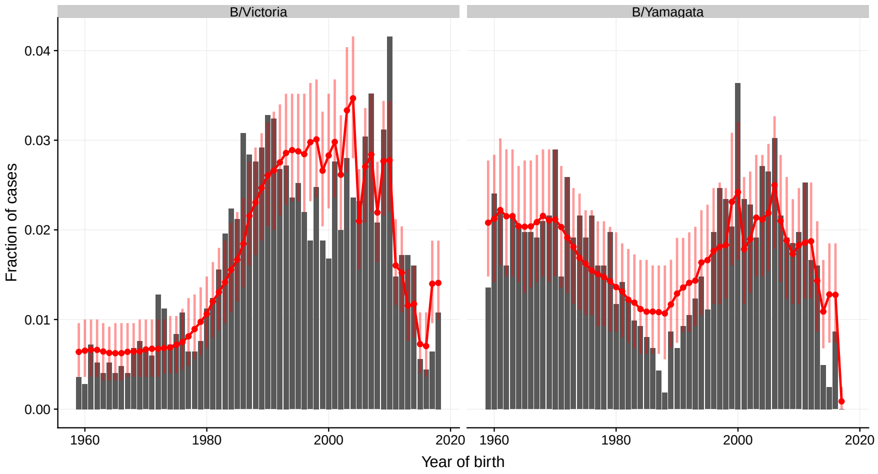

Supplementary Fig. 8. Model predictions from the sensitivity analysis of the frequency of B/Yamagata in New Zealand in the 1990s. Red lines and dots show the fraction of cases in each birth cohort as predicted by the model. Vertical bars are 95% bootstrap confidence intervals based on $n = 1000$ multinomial draws from the predicted distributions indicated by the dots.

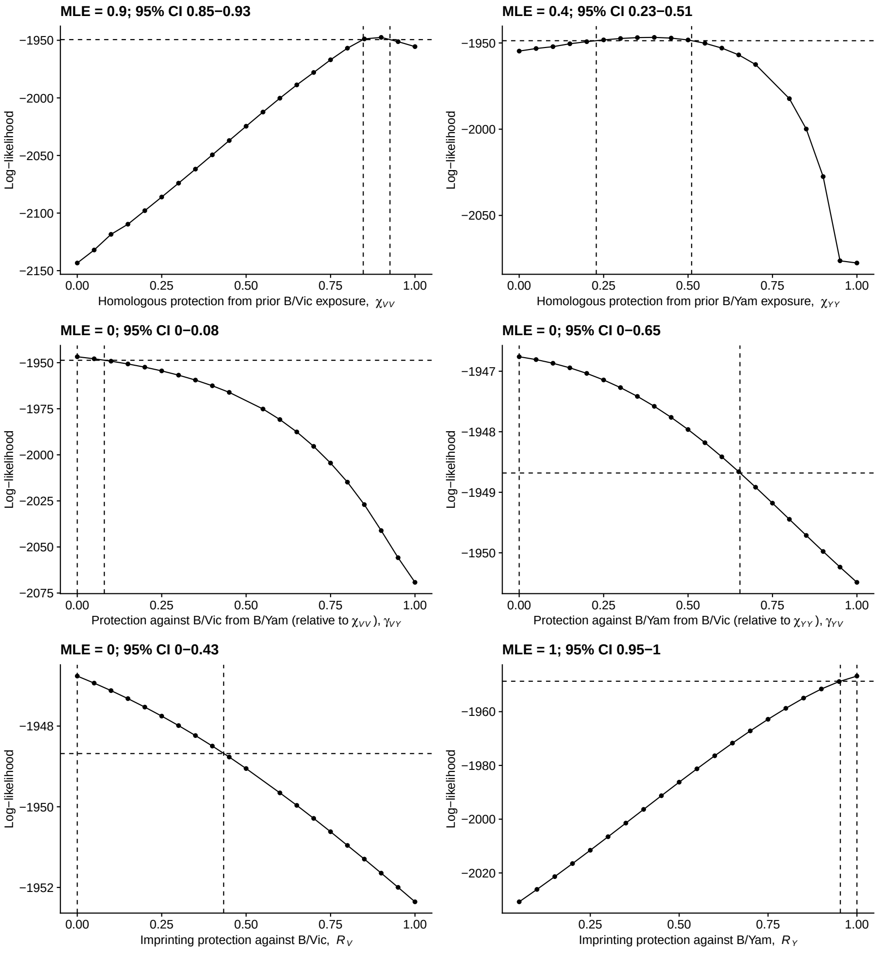

Supplementary Fig. 9. Likelihood profiles for protection parameters estimated assuming B/Victoria was the only lineage circulating between 1983 and 1990. The 95% confidence interval based on a likelihood ratio test with one degree of freedom is indicated by the vertical dashed lines.

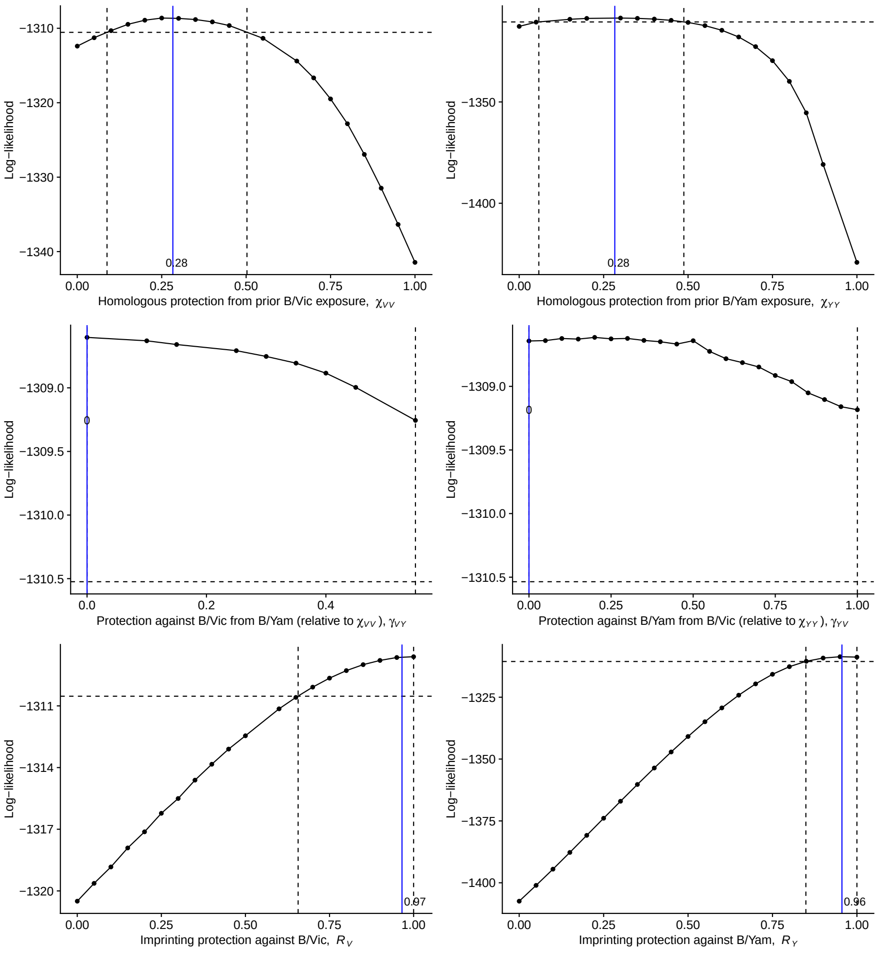

Supplementary Fig. 10. Likelihood profiles for protection parameters estimated from data simulated under strong imprinting protection but moderate within-lineage protection against B/Victoria Parameter values used for the simulation are represented by the vertical blue lines and the numbers next to them. The other parameters beside R_V and χ_{VV} were set to their maximum likelihood values from the main analysis. The 95% confidence interval based on a likelihood ratio test with one degree of freedom is indicated by the vertical dashed lines.

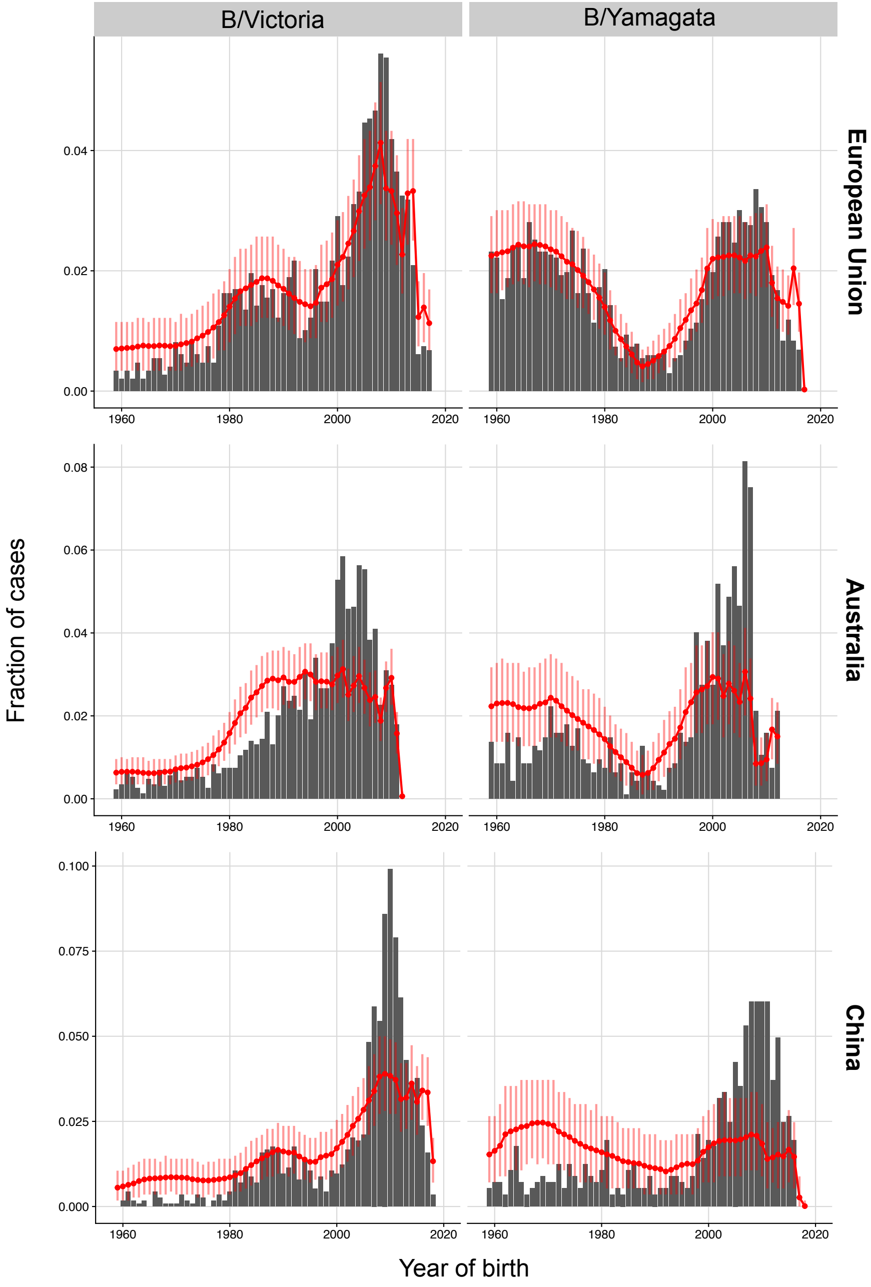

Supplementary Fig. 11. Model predictions for additional data sets using protection parameters estimated from the New Zealand data. Red lines and dots show the fraction of cases in each birth cohort as predicted by the model. Vertical bars are 95% bootstrap confidence intervals based on $n = 1000$ multinomial draws from the predicted distributions indicated by the dots.

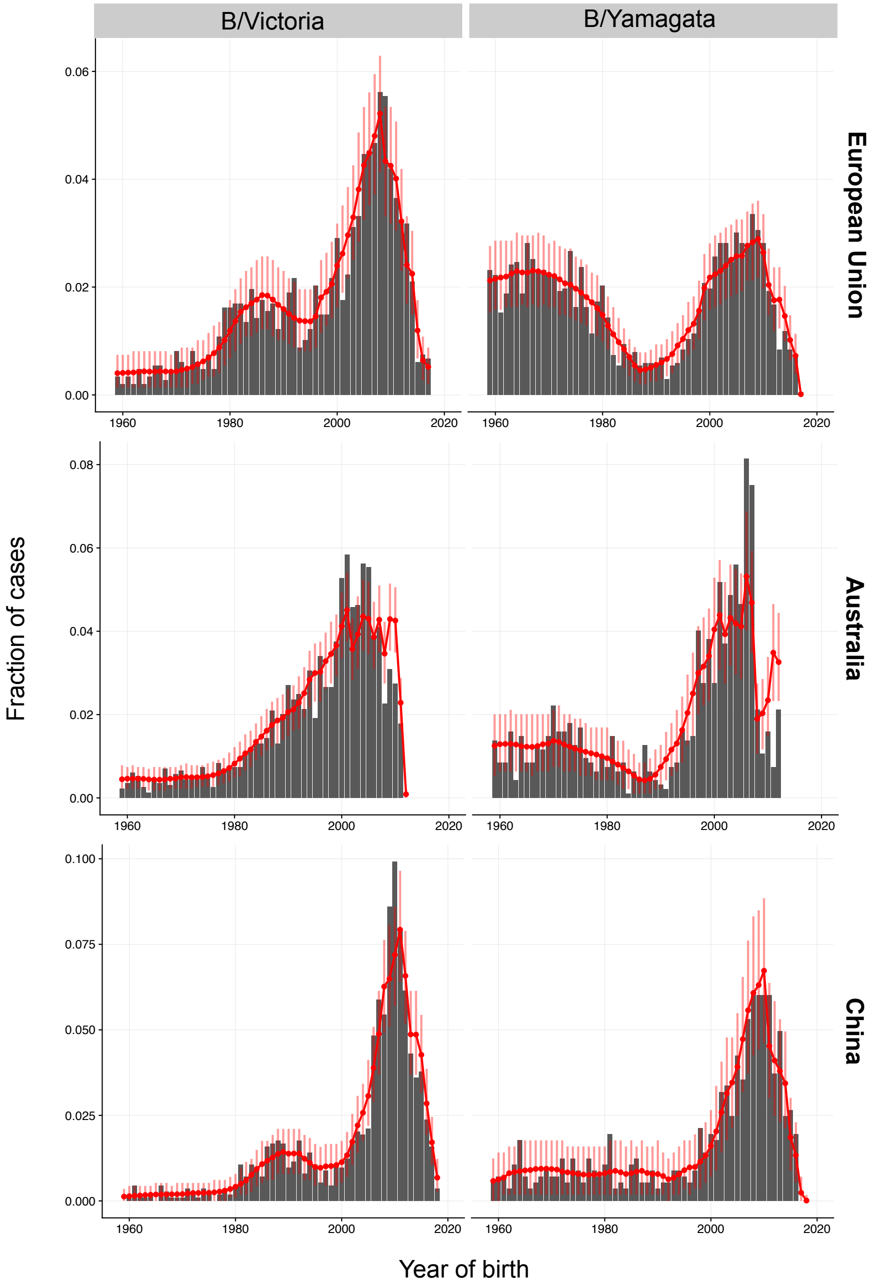

Supplementary Fig. 12. Model predictions for additional data sets using parameters re-estimated for each data set. Red lines and dots show the fraction of cases in each birth cohort as predicted by the model. Vertical bars are 95% bootstrap confidence intervals based on $n = 1000$ multinomial draws from the predicted distributions indicated by the dots.

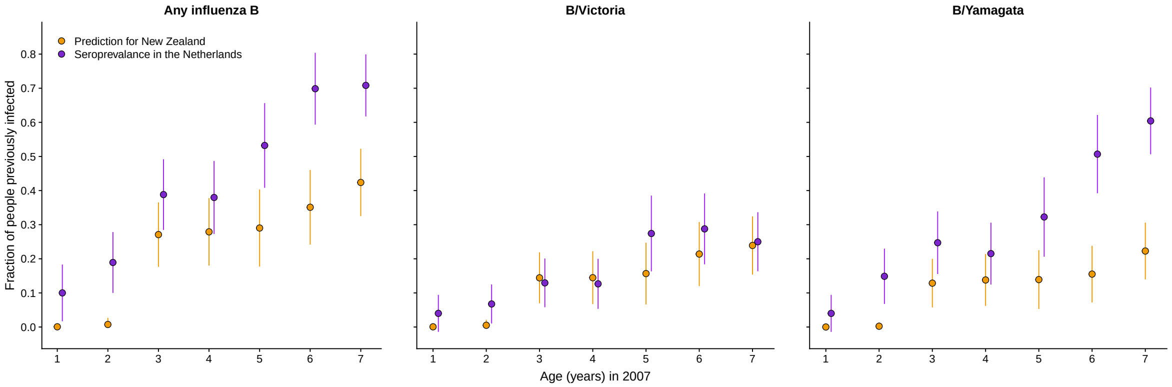

Supplementary Fig. 13. Frequency of past influenza B infections in children predicted by the model compared with observed seroprevalence in the Netherlands. The fraction of children previously infected with influenza B in the Netherlands in 2006-2007 was estimated as the fraction having serum antibodies against at least one influenza B strain from a panel of viruses (left panel), at least one B/Victoria strain (center panel) or at least one B/Yamagata strain (right panel). The circles indicate the point estimates for each age (purple) and the corresponding model predictions (orange). Fractions for Australia and New Zealand were generated independently from the seroprevalence data by fitting a statistical model to medically attended influenza B infections. Bars show 95% binomial confidence intervals. For the model predictions, binomial confidence intervals assume a sample size equal to the number of children with the corresponding age in the seroprelavence data ($n = 50, 74, 85, 79, 62, 73$ and 96 for ages 1-7, respectively).

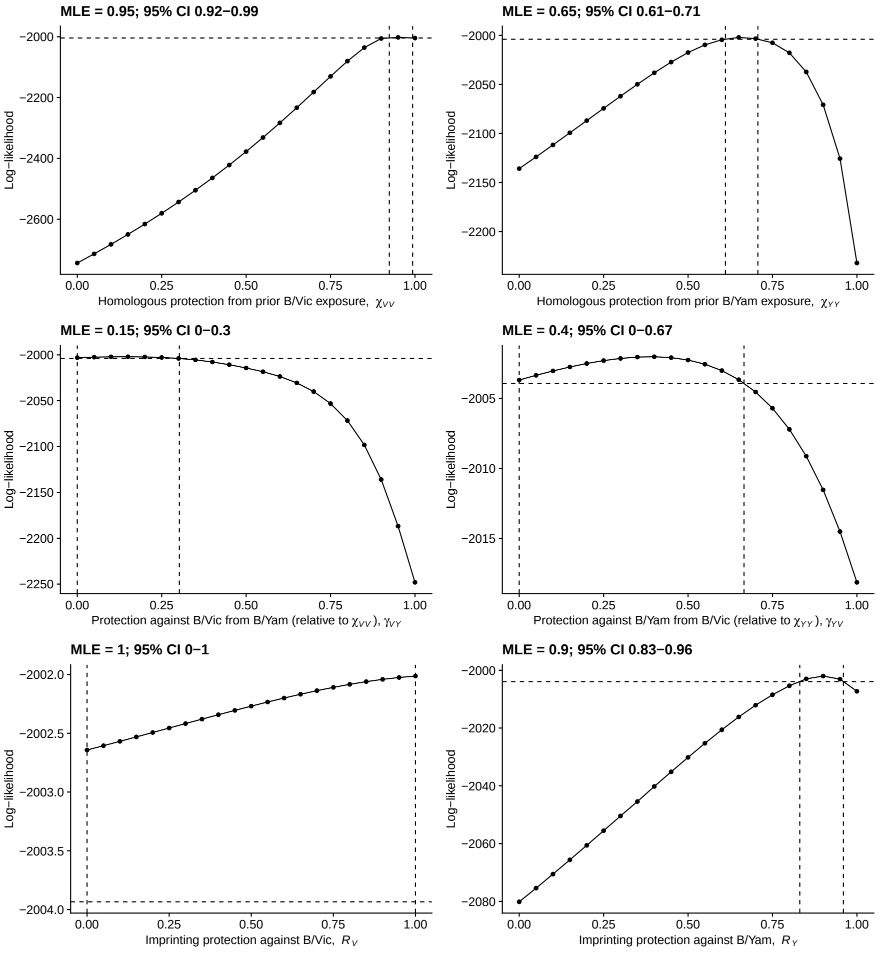

Supplementary Fig. 14. Likelihood profiles estimated under baseline annual infection probabilities constrained to values inferred from Dutch seroprevalence data. The 95% confidence interval based on a likelihood ratio test with one degree of freedom is indicated by the vertical dashed lines.

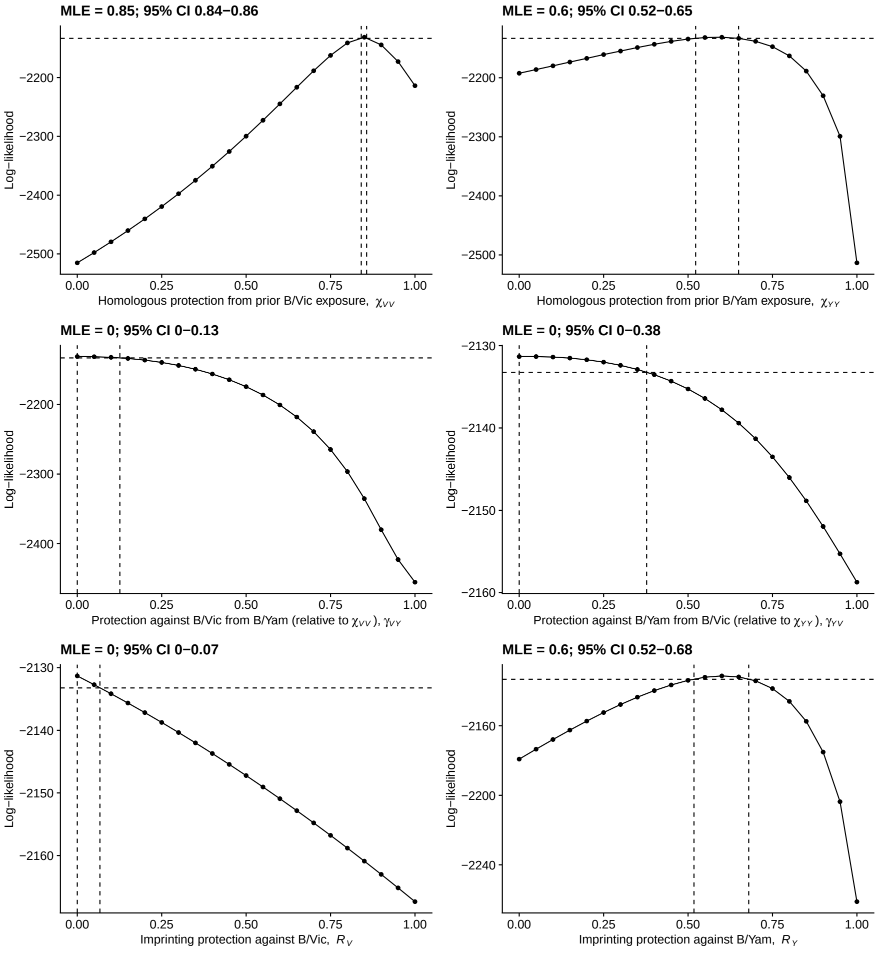

Supplementary Fig. 15. Likelihood profiles estimated under a 30% baseline annual infection probability across age groups. The 95% confidence interval based on a likelihood ratio test with one degree of freedom is indicated by the vertical dashed lines.

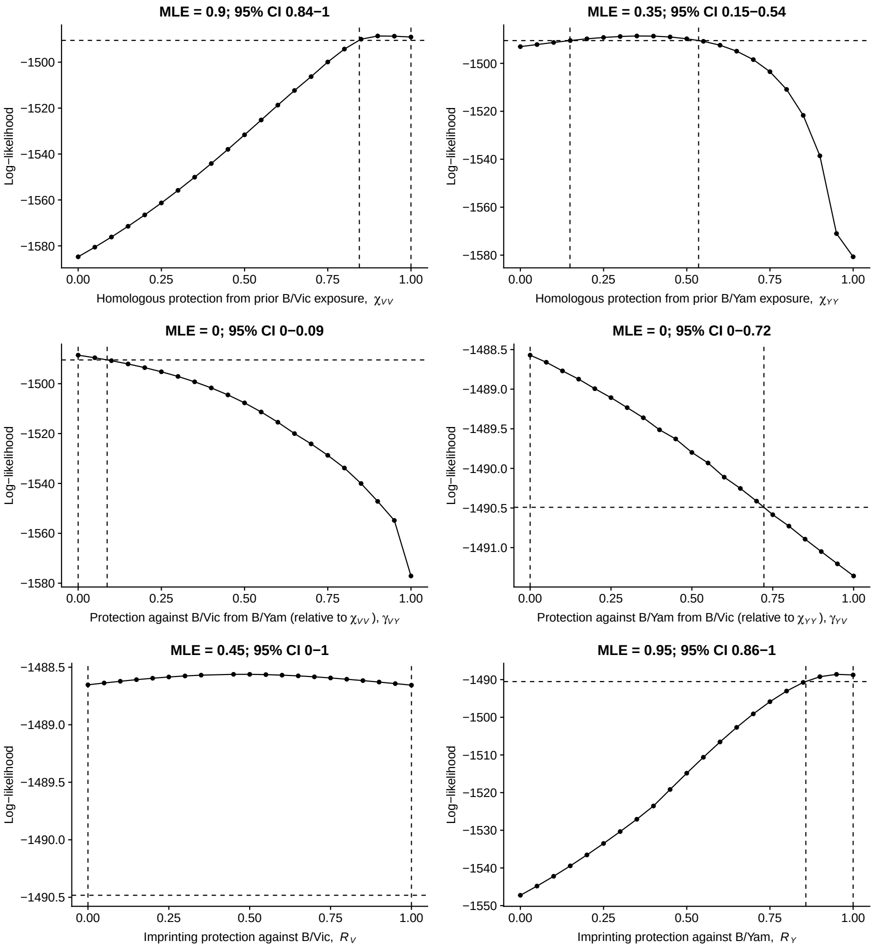

Supplementary Fig. 16. Likelihood profiles for protection parameters estimated from surveillance cases alone. The 95% confidence interval based on a likelihood ratio test with one degree of freedom is indicated by the vertical dashed lines.

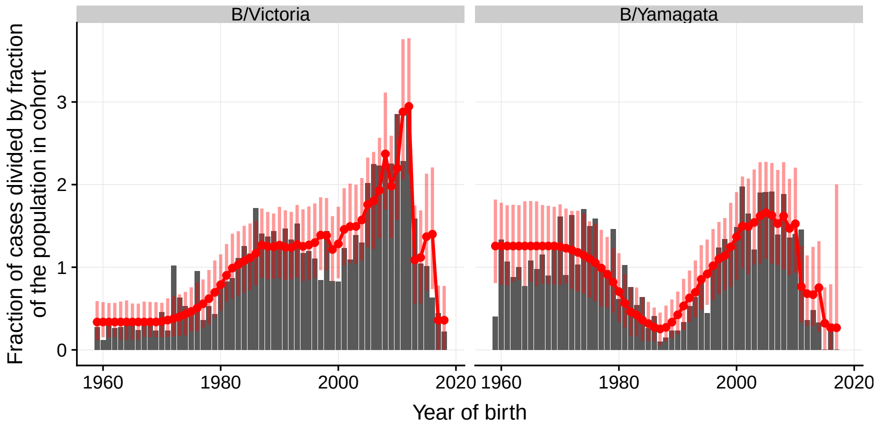

Supplementary Fig. 17. Age distributions predicted by the model fitted to surveillance cases only. Red lines and dots show the fraction of cases in each birth cohort as predicted by the model. Vertical bars are 95% bootstrap confidence intervals based on $n = 1000$ multinomial draws from the predicted distributions indicated by the dots.

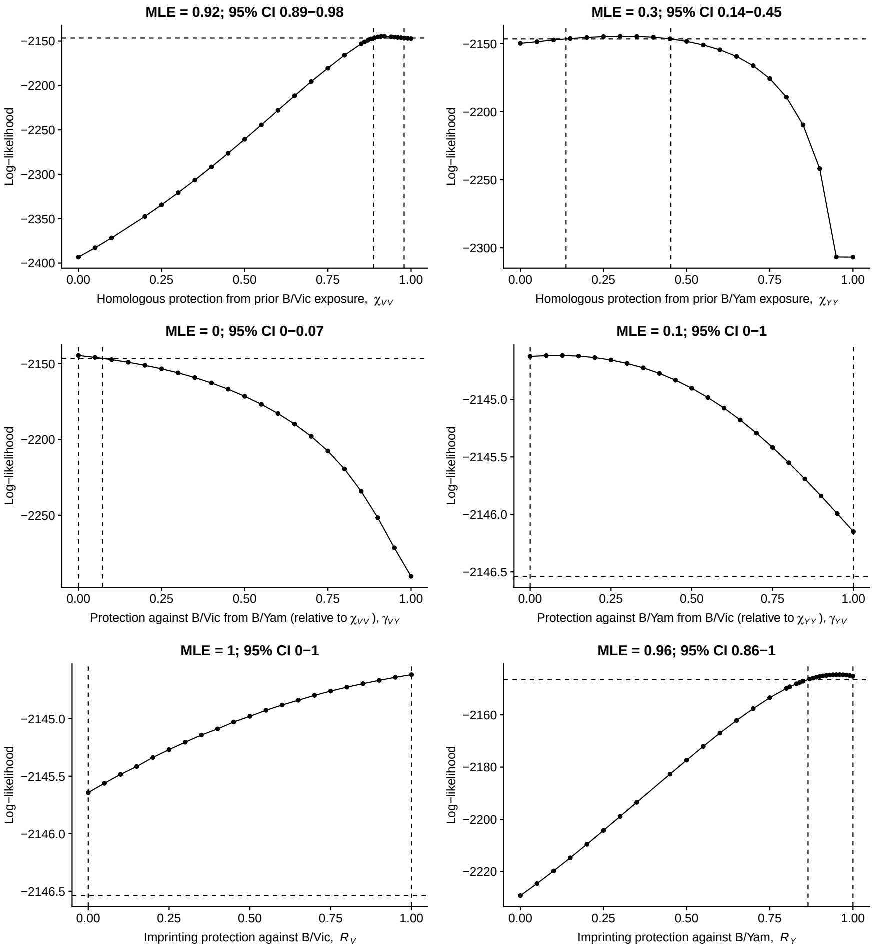

Supplementary Fig. 18. Likelihood profiles for protection parameters estimated from birth years starting in 1952 instead of 1959. The 95% confidence interval based on a likelihood ratio test with one degree of freedom is indicated by the vertical dashed lines.

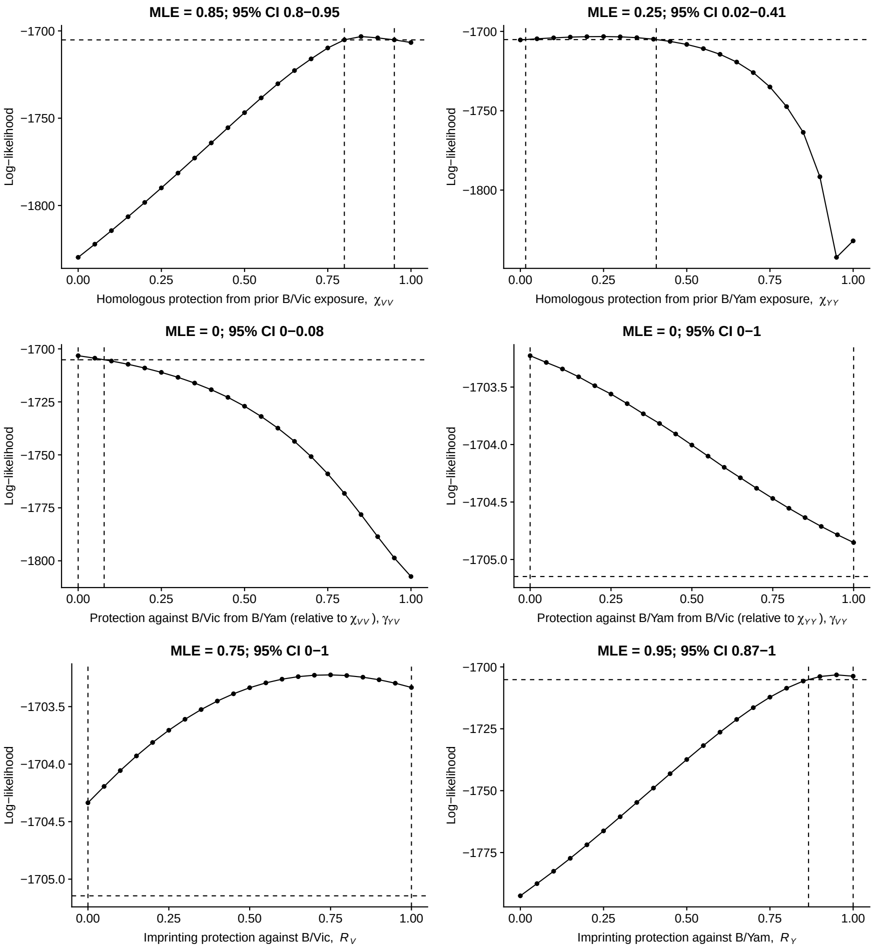

Supplementary Fig. 19. Likelihood profiles for protection parameters estimated from birth years starting in 1966 instead of 1959. The 95% confidence interval based on a likelihood ratio test with one degree of freedom is indicated by the vertical dashed lines.

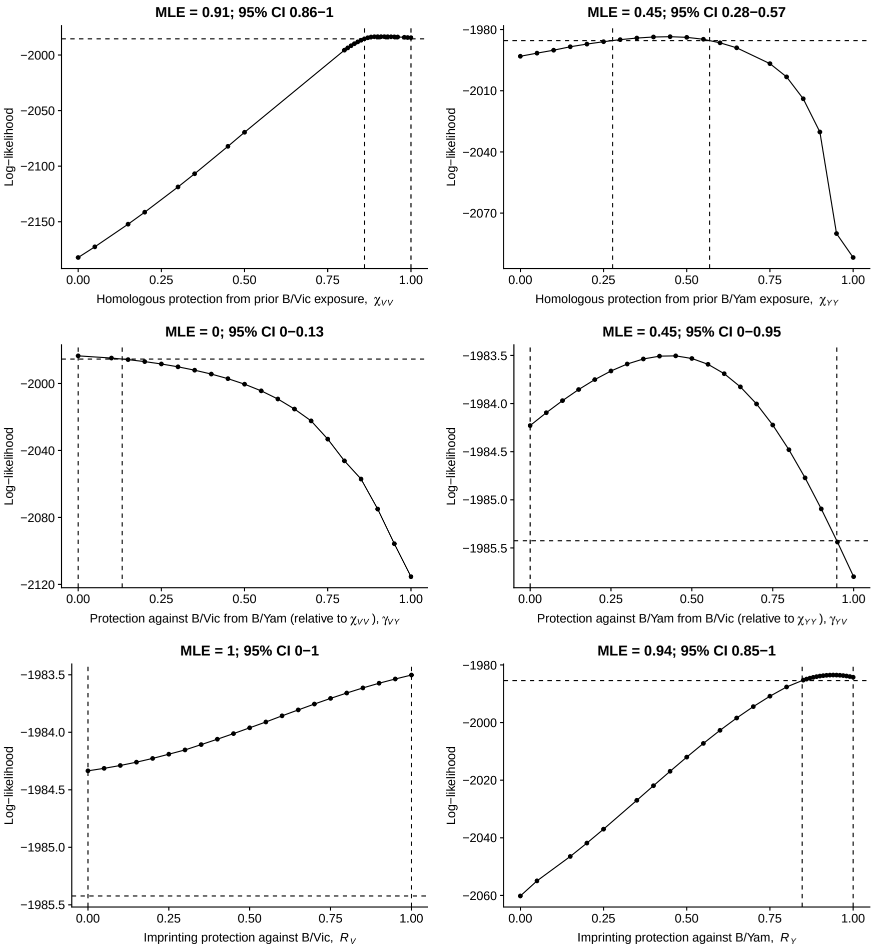

Supplementary Fig. 20. Likelihood profiles for protection parameters estimated by the model assuming a separate probability of medical attendance for infections in children under 5 years old The 95% confidence interval based on a likelihood ratio test with one degree of freedom is indicated by the vertical dashed lines.

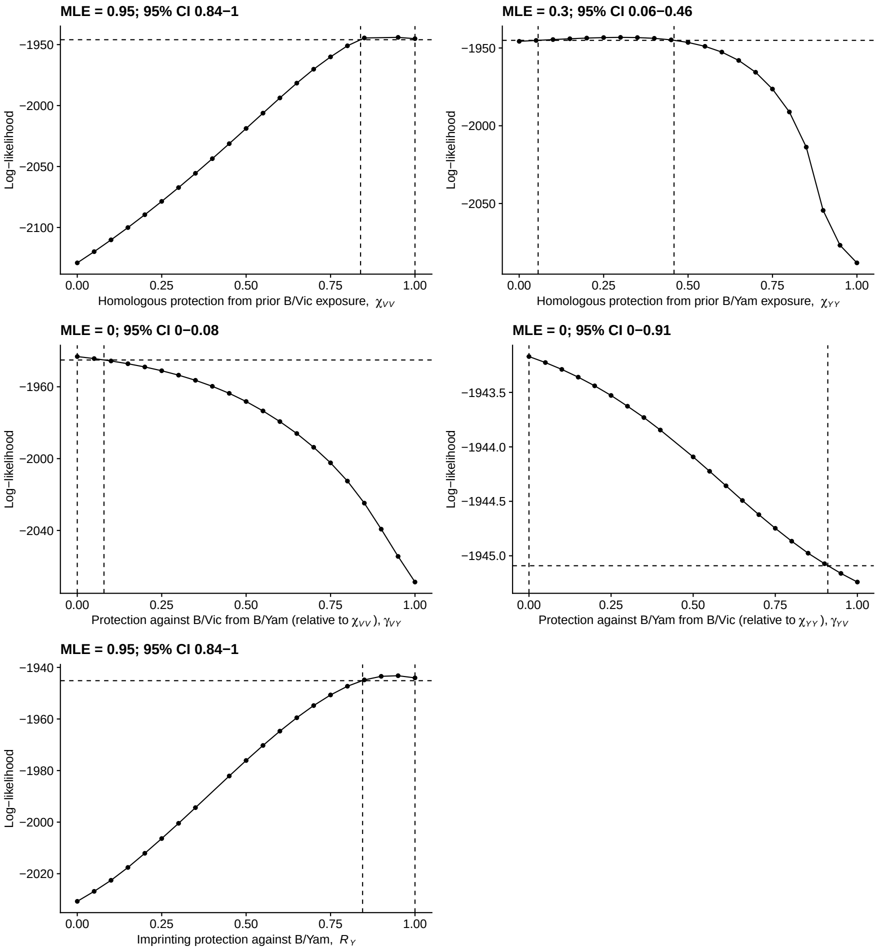

Supplementary Fig. 21. Likelihood profiles for protection parameters estimated from the complete New Zealand data after removing imprinting protection against B/Victoria and protection from strains circulating before 1988 against B/Yamagata. The 95% confidence interval based on a likelihood ratio test with one degree of freedom is indicated by the vertical dashed lines.

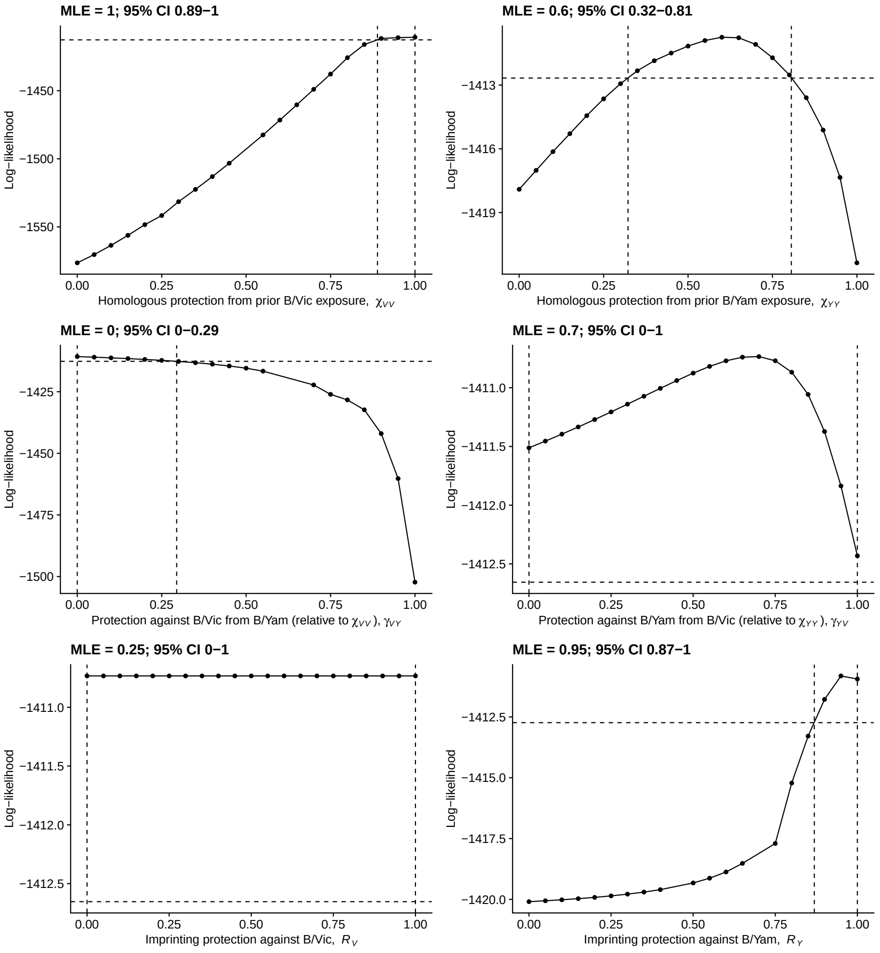

Supplementary Fig. 22. Likelihood profiles for protection parameters estimated after excluding children under 10 years old The 95% confidence interval based on a likelihood ratio test with one degree of freedom is indicated by the vertical dashed lines.

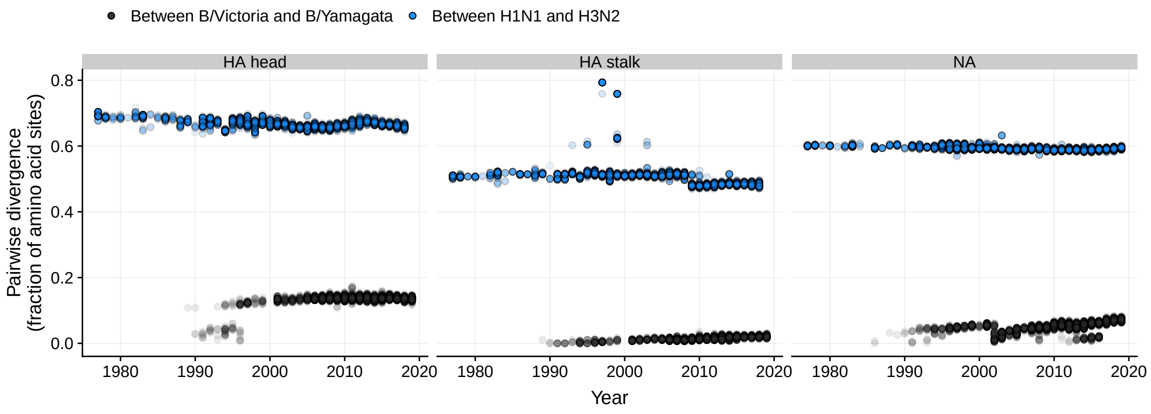

Supplementary Fig. 23. Amino acid divergence in the hemagglutinin and neuraminidase surface proteins between influenza A subtypes and between influenza B lineages. Divergence in hemagglutinin is shown separately for the antigenically variable head region (left) and the more conserved stalk region (center). Each point represents a pair of strains isolated in the same year and deposited on GISAID. When more than 100 isolates from a subtype or lineage were isolated in a single year, we used a random sample of 100 isolates, resulting in up to 4,950 pairs of each kind in each year. Up to 500 randomly chosen pairs are shown for each year. Average divergence values reported in the main text are based on the full set of pairs given the randomly sampled sequences.

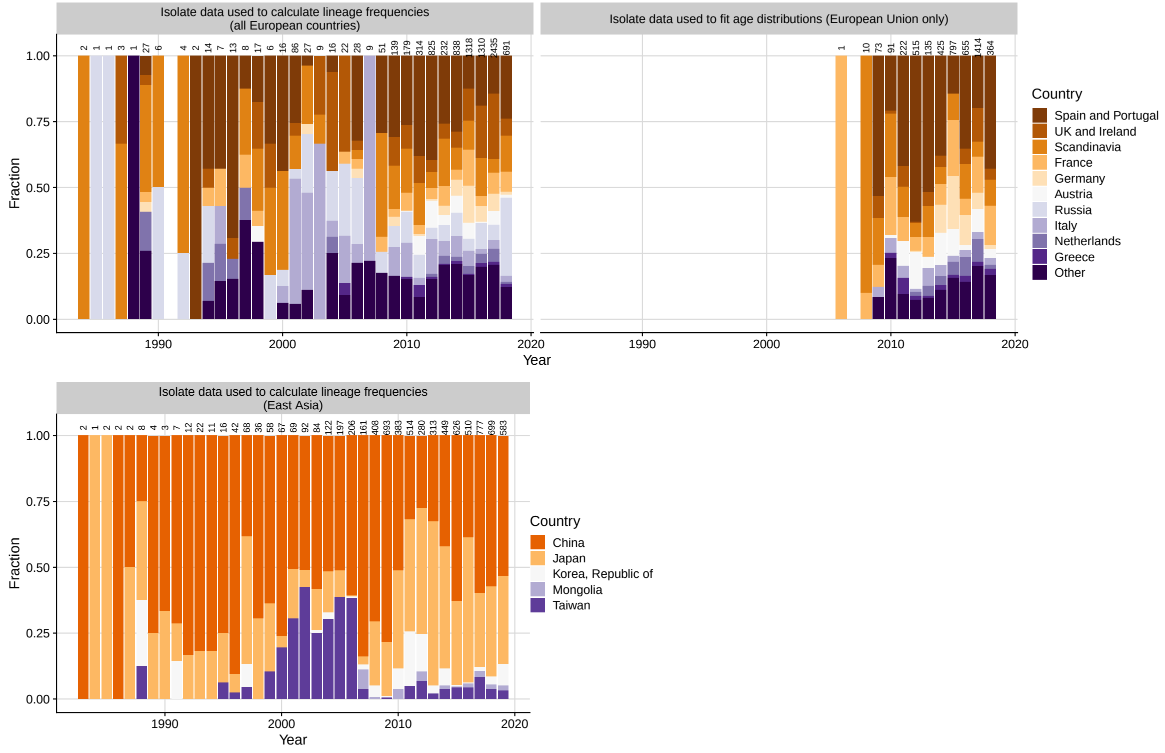

Supplementary Fig. 24. Geographic distribution of isolate data used to model the age distributions of influenza B cases in the European Union and China. We fitted the model to the age distribution of GISAID isolates in China and the European Union (EU). For those fits, historical lineage frequencies are also calculated from isolate data, including isolates that lack host age information. To increase statistical power, we used historical lineage frequencies estimated from all European countries represented on GISAID (including those not in the EU) when fitting to the EU age distributions (top left panel), and from all represented countries in East Asia when fitting to the Chinese age distributions (bottom left panel). The total numbers of isolates in each year are shown as numbers above the bars.
