# Supplementary material for: Lineage-specific protection and immune imprinting shape the age distributions of influenza B cases
Source: Nat Commun. 2021 Jul 14;12:4313. doi: 10.1038/s41467-021-24566-y (PMC8280188; doi:10.1038/s41467-021-24566-y)
Supplement: Supplementary file 2 — Reporting Summary [file 41467_2021_24566_MOESM2_ESM.pdf]

nature research

Corresponding author(s): Marcos Costa Vieira

Last updated by author(s): Jun 16, 2021

Reporting Summary

Nature Research wishes to improve the reproducibility of the work that we publish. This form provides structure for consistency and transparency in reporting. For further information on Nature Research policies, see our Editorial Policies and the Editorial Policy Checklist.

Statistics

For all statistical analyses, confirm that the following items are present in the figure legend, table legend, main text, or Methods section.

n/a	Confirmed	
☐	☒	The exact sample size (*n*) for each experimental group/condition, given as a discrete number and unit of measurement
☒	☐	A statement on whether measurements were taken from distinct samples or whether the same sample was measured repeatedly
☒	☐	The statistical test(s) used AND whether they are one- or two-sided<br>*Only common tests should be described solely by name; describe more complex techniques in the Methods section.*
☐	☒	A description of all covariates tested
☐	☒	A description of any assumptions or corrections, such as tests of normality and adjustment for multiple comparisons
☐	☒	A full description of the statistical parameters including central tendency (e.g. means) or other basic estimates (e.g. regression coefficient) AND variation (e.g. standard deviation) or associated estimates of uncertainty (e.g. confidence intervals)
☒	☐	For null hypothesis testing, the test statistic (e.g. *F*, *t*, *r*) with confidence intervals, effect sizes, degrees of freedom and *P* value noted<br>*Give P values as exact values whenever suitable.*
☒	☐	For Bayesian analysis, information on the choice of priors and Markov chain Monte Carlo settings
☒	☐	For hierarchical and complex designs, identification of the appropriate level for tests and full reporting of outcomes
☐	☒	Estimates of effect sizes (e.g. Cohen's *d*, Pearson's *r*), indicating how they were calculated

Our web collection on statistics for biologists contains articles on many of the points above.

Software and code

Policy information about availability of computer code

Data collection	No software was used to collect the data.
Data analysis	Data were analyzed using R (v 3.4.3) with packages optimParallel (v.0.7-3), FlexParamCurve (v1.5-3) and custom code available at https://doi.org/10.5281/zenodo.4969296

For manuscripts utilizing custom algorithms or software that are central to the research but not yet described in published literature, software must be made available to editors and reviewers. We strongly encourage code deposition in a community repository (e.g. GitHub). See the Nature Research guidelines for submitting code & software for further information.

Data

Policy information about availability of data

All manuscripts must include a data availability statement. This statement should provide the following information, where applicable:

- Accession codes, unique identifiers, or web links for publicly available datasets
- A list of figures that have associated raw data
- A description of any restrictions on data availability

The data used in the analyses were compiled into the GitHub repository (https://doi.org/10.5281/zenodo.4969296). The original influenza virus isolate data are available on the GISAID database (https://www.gisaid.org/) and the NCBI Influenza Virus Database (https://www.ncbi.nlm.nih.gov/genomes/FLU/Database/nph-select.cgi?go=database). Influenza surveillance reports and demographic data are available on government websites from New Zealand, Australia, China and the European Union [Links in Refs. 62, 63, 65, 66, 67 and 68 of the paper]

Field-specific reporting

Please select the one below that is the best fit for your research. If you are not sure, read the appropriate sections before making your selection.

☒ Life sciences ☐ Behavioural & social sciences ☐ Ecological, evolutionary & environmental sciences

For a reference copy of the document with all sections, see nature.com/documents/nr-reporting-summary-flat.pdf

Life sciences study design

All studies must disclose on these points even when the disclosure is negative.

Sample size	Sample size was determined by the availability of influenza sequence and case data collected by epidemiological surveillance independently of the study.
Data exclusions	To limit model complexity, we only fitted the model to cases observed in hosts born since 1959, who were 60 years old or younger at the time the data were observed. This cutoff was determined a priori, and we performed sensitivity analysis to show that the results were robust to the precise cutoff choice.
Replication	Inclusion criteria set by epidemiological surveillance independently of the study are described in the Methods. Because the data were collected for epidemiological surveillance purposes independently of the study, replication of data collection was not attempted. Reproducibility of the analyses is ensured by the public availability of the code at https://doi.org/10.5281/zenodo.4969296.
Randomization	Not applicable. The case and isolate data were collected for epidemiological surveillance purposes independently of and prior to this study. Inclusion criteria are described in the paper.
Blinding	Not applicable. The case and isolate data were collected for epidemiological surveillance purposes independently of and prior to this study. Inclusion criteria are described in the paper.

Reporting for specific materials, systems and methods

We require information from authors about some types of materials, experimental systems and methods used in many studies. Here, indicate whether each material, system or method listed is relevant to your study. If you are not sure if a list item applies to your research, read the appropriate section before selecting a response.

Materials & experimental systems

n/a	Involved in the study	
☒	☐	Antibodies
☒	☐	Eukaryotic cell lines
☒	☐	Palaeontology and archaeology
☒	☐	Animals and other organisms
☐	☒	Human research participants
☒	☐	Clinical data
☒	☐	Dual use research of concern

Methods

n/a	Involved in the study	
☒	☐	ChIP-seq
☒	☐	Flow cytometry
☒	☐	MRI-based neuroimaging

Human research participants

Policy information about studies involving human research participants

Population characteristics	We used aggregated data on influenza type B cases collected by national epidemiological surveillance systems in New Zealand and Australia or inferred from sequence isolates deposited on databases. We analyzed data aggregated by birth cohorts from 1959 to 2019. Information on gender was not generally available.
Recruitment	Influenza B cases were recorded by epidemiological surveillance systems in Australia and New Zealand in individuals presenting to general practitioners with influenza-like illness or in individuals hospitalized with acute respiratory infections. Case definitions and inclusion criteria are detailed in the Methods. Because only individuals who sought medical attention had a chance of being reported as a case that was later included in the data, our results only reflect a subset of all influenza B infections. We could not estimate protection against sub-clinical influenza infections or paucisymptomatic infections that do not result in healthcare seeking.
Ethics oversight	Not applicable. Only aggregated data previously collected by epidemiological surveillance were used in the analyses.

Note that full information on the approval of the study protocol must also be provided in the manuscript.
